# In vivo quantitative assessment of therapeutic response to bortezomib therapy in disseminated animal models of multiple myeloma with [^18^F]FDG and [^64^Cu]Cu-LLP2A PET

**DOI:** 10.1186/s13550-021-00840-4

**Published:** 2021-09-29

**Authors:** Anchal Ghai, Nikki Fettig, Francesca Fontana, John DiPersio, Mike Rettig, Julie O. Neal, Samuel Achilefu, Kooresh I. Shoghi, Monica Shokeen

**Affiliations:** 1 Department of Radiology, Mallinckrodt Institute of Radiology, Washington University School of Medicine, 4515 McKinley Avenue, 2nd floor, St. Louis, MO 63110 USA; 2 Department of Internal Medicine, Washington University School of Medicine, St. Louis, MO USA; 3 Department of Medicine, Washington University School of Medicine, St. Louis, MO USA; 4 Department of Biomedical Engineering, Washington University in St. Louis, St. Louis, MO USA; 5 Department of Biochemistry and Molecular Biophysics, Washington University School of Medicine, St. Louis, MO USA

**Keywords:** Therapy response, Very late antigen-4 (VLA4), [^64^Cu]Cu-LLP2A, [^18^F]FDG, Multiple myeloma (MM), Bortezomib (proteasome inhibitor) therapy

## Abstract

**Background:**

Multiple myeloma (MM) is a disease of cancerous plasma cells in the bone marrow. Imaging-based timely determination of therapeutic response is critical for improving outcomes in MM patients. Very late antigen-4 (VLA4, CD49d/CD29) is overexpressed in MM cells. Here, we evaluated [^18^F]FDG and VLA4 targeted [^64^Cu]Cu-LLP2A for quantitative PET imaging in disseminated MM models of variable VLA4 expression, following bortezomib therapy.

**Methods:**

In vitro and ex vivo VLA4 expression was evaluated by flow cytometry. Human MM cells, MM.1S-CG and U266-CG (C: luciferase and G: green fluorescent protein), were injected intravenously in NOD-SCID gamma mice. Tumor progression was monitored by bioluminescence imaging (BLI). Treatment group received bortezomib (1 mg/kg, twice/week) intraperitoneally. All cohorts (treated, untreated and no tumor) were longitudinally imaged with [^18^F]FDG (7.4–8.0 MBq) and [^64^Cu]Cu-LLP2A (2–3 MBq; Molar Activity: 44.14 ± 1.40 MBq/nmol) PET, respectively.

**Results:**

Flow cytometry confirmed high expression of CD49d in U266 cells (> 99%) and moderate expression in MM.1S cells (~ 52%). BLI showed decrease in total body flux in treated mice. In MM.1S-CG untreated versus treated mice, [^64^Cu]Cu-LLP2A localized with a significantly higher SUV_mean_ in spine (0.58 versus 0.31, *p* < 0.01) and femur (0.72 versus 0.39, *p* < 0.05) at week 4 post-tumor inoculation. There was a four-fold higher uptake of [^64^Cu]Cu-LLP2A (SUV_mean_) in untreated U266-CG mice compared to treated mice at 3 weeks post-treatment. Compared to [^64^Cu]Cu-LLP2A, [^18^F]FDG PET detected treatment-related changes at later time points.

**Conclusion:**

[^64^Cu]Cu-LLP2A is a promising tracer for timely in vivo assessment of therapeutic response in disseminated models of MM.

**Supplementary Information:**

The online version contains supplementary material available at 10.1186/s13550-021-00840-4.

## Background

Multiple myeloma (MM) is a cancer of abnormal plasma cells in the bone marrow. It is a complex disease characterized by intratumoral heterogeneity, inflammatory tumor microenvironment, and genetic instability [1, 2]. Conventional methods for diagnosing MM include complete blood count (CBC), protein electrophoresis (urine and serum) with immunofixation, nephelometric quantitation of immunoglobulins and serum analysis for evaluating calcium and creatinine levels. Bone marrow testing is done with aspirate and trephine biopsy for cytogenetics and determination of cancerous plasma cells. Finally, bone survey is almost always performed, while other advanced imaging methods are used on a case by case basis [3]. Whole-body computed tomography (CT) and magnetic resonance imaging (MRI) are effective modalities for detecting osteolytic bone and focal bone marrow lesions [4–6]. Patients with MM are showing significantly improved survival rates due to improved diagnostics, and successful transplantation and evolving therapies; however, most patients relapse with refractory disease [7]. There remains an urgent need to develop techniques for timely identification of patients whose disease is progressing aggressively and patients who could benefit from a change in their therapy [8, 9]. Prognostic classification of patients solely based on genetics can be challenging at the time of diagnosis due to issues such as inadequate sampling. Therefore, monitoring of therapeutic response is considered an important approach to identify patients who are progressing quicker and should be monitored closely [10]. Furthermore, the International Myeloma Working Group (IMWG) has proposed the evaluation of minimal residual disease (MRD) as one of the key criteria for defining response [11]. Techniques such as whole-body imaging that can improve the accuracy of MRD detection are therefore critical for comprehensive assessment of complete response [12].

Positron emission tomography (PET) is a positron-emitting radionuclide based functional imaging technique that is highly sensitive for detecting disease burden of varying degree [13]. Molecularly targeted PET radiopharmaceuticals have been successfully utilized for assessing biological processes at cellular and molecular levels, enabling early and accurate identification of cancerous cells and the enabling tumor microenvironment [14]. Multiple myeloma (MM) is a radiologically defined disease [15]. PET fused with advanced anatomic imaging modalities, such as CT and MRI, provides a whole-body, high-resolution and sensitive platform for probing the biology of disease, achieving early diagnosis, improved disease staging, treatment planning and evaluation of therapeutic response [16, 17]. PET/CT or PET/MR imaging facilitates longitudinal and sensitive detection of myeloma induced bone lesions, bone marrow infiltration, compositional changes in the microenvironment, and extra medullary disease [18, 19]. [^18^F]FDG is a radiolabeled glucose analog that helps measure glucose metabolism. Mimicking glucose molecule, [^18^F]FDG gets internalized into the cells via glucose transporter protein 1 (GLUT1) and metabolized intracellularly by hexokinase 2 enzyme; both molecules generally overexpressed in tumor cells. The field of hematological imaging has traditionally been dominated by [^18^F]FDG and to a very good effect. In fact, [^18^F]FDG is the most utilized metabolic PET imaging tracer to assess therapy response in patients with hematological malignancies like lymphoma [20–22] and MM [23, 24]. However, despite being the PET gold standard for MM imaging, [^18^F]FDG has certain limitations in MM. [^18^F]FDG is unable to distinguish between malignant cells and inflammation following therapy (flare reaction to steroids), leading to false positive results [25, 26]. Contrarily, in metabolically low MM lesions, [^18^F]FDG PET invariably results in underestimation of tumor burden [26]. Rashe et al. showed that low expression of hexokinase-2 is associated with false-negative FDG PET in MM [27]. [^18^F]FDG PET also has less than optimal efficacy for detecting diffuse bone marrow infiltration in patients with MM [24]. To overcome these limitations, a MM specific and sensitive PET imaging agent is desirable in MM patients.

Recently, there have been considerable advancements in the development of molecularly targeted PET imaging agents that are closer to clinic than ever before [28, 29]. Molecular imaging of specific proteins overexpressed in tumor cells will be key to realizing the concept of precision health [30]. As promising new tracers are getting traction in clinic, their evaluation relative to the existing gold standard contrast agents can provide pertinent information regarding the unique and complementary data that is rendered by these agents. Very late antigen-4 (VLA4; also known as integrin α_4_β_1_, CD49d/CD29) is a non-covalent, heterodimeric integrin receptor that is upregulated in MM [31]. Scientific literature strongly supports the role of VLA4 in tumor proliferation and metastasis [32, 33]. It also plays a crucial role in drug resistance (cell adhesion mediated drug resistance (CAM-DR)) in hematological malignancies such as MM [34] and acute myelogenous leukemia [33].

[^64^Cu]Cu-LLP2A is a VLA4 targeted, high-affinity radiopharmaceutical. We and others have shown the efficacy of [^64^Cu]Cu-LLP2A in different pathologies including MM and melanoma [31, 35–38]. There is currently an ongoing clinical trial to assess the safety and dosimetry of [^64^Cu]Cu-LLP2A in healthy and MM patient volunteers [39]. Given the widespread use of [^18^F]FDG in MM, there is strong rationale for investigating the unique qualitative and quantitative features of these tracers in a medullary myeloma setting.

In this study, we compared the efficacy of [^18^F]FDG PET with VLA4 targeted [^64^Cu]Cu-LLP2A PET via longitudinal imaging of disease progression in disseminated preclinical models of human myeloma. We additionally performed a head-on comparison of these tracers following bortezomib therapy in the same model. Bortezomib is a FDA approved reversible proteasome inhibitor, widely used either alone or in combination with other agents for treating MM [40]. Bortezomib therapy is considered an effective treatment regimen for MM [41, 42]. Studies have shown that bortezomib can downregulate VLA4 expression in myeloma cells and help reduce CAM-DR [43]. Additionally, recent work demonstrates that bortezomib-refractory myeloma cells have higher VLA4 expression as compared to the parental cells [44].

We utilized two human models of disseminated myeloma, expressing different levels of VLA4 protein for assessing tracer performance in vivo*.* Standard uptake values (SUV) from PET data provide a reliable semi-quantitative measure of the tumor uptake and kinetics in various tissues and help assess therapy response [45, 46]. Here, the radiotracer uptake was quantified (via SUV) in a longitudinal setting (weekly sequential imaging with both tracers), and complemented with bioluminescence imaging (BLI) and ex vivo flow cytometry, with a focus on the intramedullary disease burden.

In summary, we evaluated the quantitative and qualitative features of these two promising PET tracers in MM models. The underlying hypothesis is that molecular imaging of plasma cell receptor VLA4 can provide promising synergistic and timely information on disease progression and therapeutic response.

## Material and methods

### Ethics statement

All the experiments involving the use of radioactive materials were done at Washington University and conducted under the authorization of the Radiation Safety Commission in accordance with the University’s Nuclear Regulatory Commission license. All animal studies were performed in accordance with the Guide for the Care and Use of Laboratory Animals under the auspices of the Animal Studies Committee of Washington University (Animal Welfare Assurance number – D16-00245).

### Reagents

Chemicals and reagents used in the present study were of highest commercially available purity, and all the solutions were prepared using ultrapure water (18 MV-cm resistivity; Millipore system). The proteasome inhibitor, bortezomib, was purchased from Sigma-Aldrich. LLP2A-CB-TE1A1P (LLP2A) peptide was purchased from Auspep (Tullamarine Victoria, Australia), and all other chemicals used in radiolabeling were purchased from Sigma-Aldrich unless otherwise noted. Copper 64 (*t*_1/2_—12.7 h) was produced on a CS-15 biomedical cyclotron at Washington University School of Medicine. Radiochemical purity of the labeled peptide was evaluated by analytical reversed-phase high-performance liquid chromatography (HPLC), that was performed on 1200 Infinity series chromatography system, purchased from Agilent Technologies (Santa Clara, CA). The XB-C18 Kinetex column which was procured from Phenomenex was used with mobile phases of 0.1% TFA in water (aqueous phase) and 0.1% TFA in acetonitrile (organic phase). [^18^F]FDG was produced in compliance with good manufacturing practices (GMP) by Washington University Cyclotron facility.

### Cell culture

The human myeloma cell lines, MM.1S and U266, were obtained from Professor Katherine N. Weilbaecher (Department of Medicine, Washington University School of Medicine) and American Tissue Culture Collection (ATCC), respectively. MM.1S and U266 cell lines were modified to carry click beetle red luciferase (CBR; C) and green fluorescent protein (GFP; G) by Professor John DiPersio’s group (Department of Internal Medicine, Bone Marrow Transplant Division, Washington University School of Medicine). The cells were maintained in suspension at 10^6^ cells/mL in complete Roswell Park Memorial Institute (RPMI) 1640 medium (Thermo Fischer Scientific). The RPMI media was supplemented with 10% fetal bovine serum (Gibco) and 1% penicillin/streptomycin (Thermo Fischer Scientific). The cells were cultured in a jacketed humidified CO_2_ (5%) incubator at 37 °C and passaged when they were confluent. Both the cell lines were tested for mycoplasma contamination and were negative for mycoplasma.

### MM.1S-CG/NSG and U266-CG/NSG MM mouse models

NOD-SCID gamma (NSG) mice purchased from Jackson laboratories (USA) were used to develop the MM mouse models utilized in this study. Mice were housed in ventilated cages and allowed food and water. MM.1S-CG (5e^6^ cells in 100 µL) and U266-CG (20e^6^ cells in 100 µL) cells were injected into the NSG mice via tail vein to establish disseminated MM disease. Tumors were allowed to grow for 1–2 weeks before initiation of bortezomib therapy. Tumor progression was monitored by weekly bioluminescent imaging (BLI). All tumor inoculation and imaging procedures were conducted under isoflurane anesthesia (1–2% vaporized in O_2_).

### Bioluminescent imaging (BLI)

**Fig. 1:**
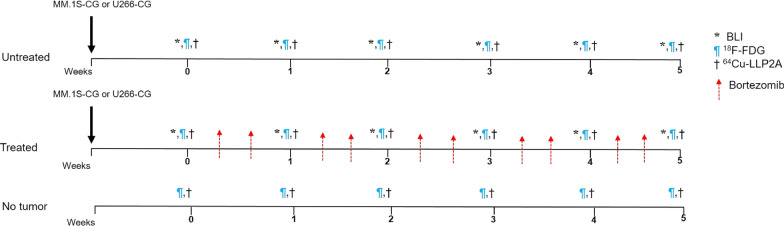
Imaging and therapy study design. NOD-SCID Gamma (NSG) mice were injected with human myeloma cell lines, MM.1S-CG and U266-CG via tail vein. Tumor progression was monitored weekly by bioluminescence imaging (BLI). Mice were divided into three cohorts—tumor bearing untreated (*n* = 6), tumor bearing treated (*n* = 6) and no tumor healthy mice (*n* = 4). The treatment group was injected with bortezomib (1 mg/kg) twice a week intraperitoneally starting at week 1 post-inoculation of MM.1S-CG and U266-CG cells, respectively. Mice were imaged weekly with small animal [^18^F]FDG PET and [^64^Cu]Cu-LLP2A PET, respectively

The tumor progression in vivo was monitored by BLI using Caliper IVIS Imager (PerkinElmer, Waltham, MA, USA; Living Image 3.2, 1–300 s exposures, binning 2–8, FOV 12.5 cm). Mice were injected with 150 mg/kg d-luciferin in PBS intraperitoneally and imaged 10 min post-injection under isoflurane anesthesia. The total photon flux (photons/sec) was measured from regions of interest (ROIs) over the entire dorsal/ventral side of the mouse, using LivingImage 2.6 (Xenogen, CA. U.S.A). The optical signal was normalized to average radiance expressed in photons per second per centimeter square per steradian (p/s/cm^2^/sr). Once the total body flux from each mouse reached 1e^6^ p/s/cm^2^/sr, longitudinal PET imaging was initiated followed by bortezomib therapy (Fig. 1). The first PET imaging time point was considered baseline for all the cohorts, including the therapy cohort (pre-therapy imaging time point, week 0).

### Bortezomib therapy

NSG mice bearing MM.1S-CG and U266-CG tumors and non-tumor bearing control mice were divided into three groups: no tumor (control mice with no tumor burden; *n* = 4), untreated (tumor bearing, no treatment; *n* = 6) and treated (tumor bearing, bortezomib treatment; *n* = 6). Bortezomib was reconstituted in 0.9% saline and was administered within 2–3 h of reconstitution [47]. Mice bearing MM tumors (treatment cohort) were injected with 1 mg/kg of bortezomib (in 0.9% saline) intraperitoneally twice a week [48–51]. The treatment began at week 1 (relative to the week 0 baseline imaging time point) (Fig. 1). The study design ensured that each mouse is its own control. The disease burden and therapy response was independently evaluated by quantification of luciferase signal in vivo (BLI imaging).

### [^64^Cu] labeling of LLP2A-CB-TE1A1P (LLP2A)

LLP2A peptide was radiolabeled with [^64^Cu] as previously described [31]. Briefly, [^64^Cu]Cu-chloride was diluted with 0.1 M ammonium acetate solution (pH 5.5). LLP2A (2.5 µg; 1.61 nmol) was incubated with 74 MBq of [^64^Cu] at 70 °C for 30–40 min with shaking. After the incubation, the radiochemical yield and purity were determined by analytical radio-high-performance liquid chromatography (HPLC) of the crude product.

### Small animal PET imaging and image analysis

No-tumor control, and MM.1S-CG/U266-CG myeloma tumor bearing (treated and untreated) mice were imaged with [^18^F]FDG and [^64^Cu]Cu-LLP2A PET/CT at baseline (week 0) and then sequentially every week for up to 6 weeks. While we were able to image a sub-set of treated mice up to 6 weeks, the mice in other two cohorts either died earlier due to disease burden or stress due to multiple imaging sessions[52]. Survival plot is included in the supplementary information (Additional file 1: Fig. S2). In preparation for the [^18^F]FDG PET imaging session, mice were fasted for 4 h with access only to water. Prior to the [^18^F]FDG PET imaging session, mice were first injected with 7.4–8 MBq via tail vein and imaged for 10 min at 1 h post-injection of the radiotracer on the small animal INVEON PET/CT scanner (Siemens Medical Solutions, Knoxville, TN). Next day after the radioactivity from [^18^F]FDG had decayed, the same group of mice was injected with the VLA4 targeted radiotracer, [^64^Cu]Cu-LLP2A (2–3 MBq) via tail vein. Whole-body small animal PET/CT static imaging (20 min static) was performed at 4 h post-injection. Images were reconstructed using a 2DOSEM algorithm. Computed tomography (CT) and corresponding PET images were co-registered on Inveon Research Workplace (IRW) software (Siemens Medical Solutions, Knoxville, TN). The reconstructed PET/CT images were viewed on IRW software, which allowed trans-axial, coronal, and sagittal displays of the slices and maximum intensity projection (MIP) PET/CT images. The volumetric regions of interest (ROI) were manually drawn using a 2-dimensional tool on the sagittal attenuation-corrected (using CT anatomical guidelines) PET slices. A semi-quantitative analysis of [^18^F]FDG and [^64^Cu]Cu-LLP2A activity was performed by calculating the mean standard uptake values (SUV_mean_) within a ROI using the formula: SUV = (*A* [nCi/mL] × [weight (g)/[dose (nCi)]) where *A* is the average activity in nCi/mL, in the specified volume of interest and decay corrected to the scan start time. Dose is the activity injected in nCi at the injection time, decay corrected to the imaging time, and weight is the whole animal in grams. Supplementary file includes additional details about the ROIs (Additional file 1: Fig. S3).

### In vitro and ex vivo flow cytometry

The human myeloma cell lines MM.1S and U266 without any reporters were analyzed for CD49d (α4 subunit of VLA4, α_4_β_1_) expression by flow cytometry. In preparation for cell surface staining, cells were suspended in 100 µL buffered (pH ~ 7.4) phosphate buffered saline (PBS) containing 0.1% bovine serum albumin (BSA). Cells were incubated with the phycoerythin-cyanine 5 conjugated anti-CD49d antibody or isotype control antibody for 30 min at 4 °C in dark. After the incubation, cells were washed twice with 1 X PBS buffer and analyzed on a FACS Calibur 3 system (BD Biosciences). Data were analyzed using FlowJo software (BD Biosciences, San Jose, CA, USA).

To evaluate VLA4 expression ex vivo, cells were extracted by flushing the bone marrow from tibia, femur and pelvis of MM.1S-CG and U266-CG IV tumor bearing mice. Utmost care was taken to preserve the viability of the cells. Briefly, cells were washed with fluorescence-activated cell sorting (FACS) buffer (1 X PBS, 0.5 M EDTA, and 0.5% BSA), stained, and immediately analyzed using LSR Fortessa (BD Biosciences). The cells were stained for human CD29 (β_1_) (APC anti-human CD29 antibody; BioLegend), mouse CD45 (BV510 Rat anti-mouse CD45; BD Biosciences), and human CD49d (PE mouse anti-human CD49d antibody; BD Biosciences) and incubated for 30 min at 4 °C in the dark. 7-Aminoactinomycin D (Thermo Fischer Scientific) (7AAD)^−^ /GFP^+^ population was considered viable tumor cells. Blue laser (Ex. 488 nm) was used to detect 7AAD (Em. 695/40 nm) and GFP (Em. 530/30 nm), red laser (Ex. 640 nm) was used to detect APC anti-CD29 (Em. 670/30) while violet laser (Ex. 405 nm) was used to detect BV510 anti-CD45 (Em. 525/50). PE anti-CD49d (Em. 585/15) was detected by yellow/green laser (Ex. 552 nm). Flow cytometry data were analyzed with FlowJo software (BD, San Jose, CA, USA).

### Statistical analysis

All the data are presented as mean ± standard deviation. Statistical analysis was performed by GraphPad Prism 8.0 (GraphPad Software, Inc., La Jolla, CA, USA). Statistical significance between cohorts was calculated using one-way ANOVA followed by Tukey’s multiple comparison tests. *p* values less than 0.05 were considered statistically significant.

## Results

### [^64^Cu] labeling of LLP2A-CB-TE1A1P

The VLA4 targeted peptide, LLP2A-CB-TE1A1P (LLP2A), was successfully radiolabeled as previously described with [^64^Cu], resulting in a molar activity of 44.14 ± 1.40 MBq/nmol [31]. The radiochemical purity of > 99 ± 0.03% was confirmed by analytical radio-HPLC (Additional file 1: Fig. S1).

### Bioluminescent imaging (BLI)

**Fig. 2:**
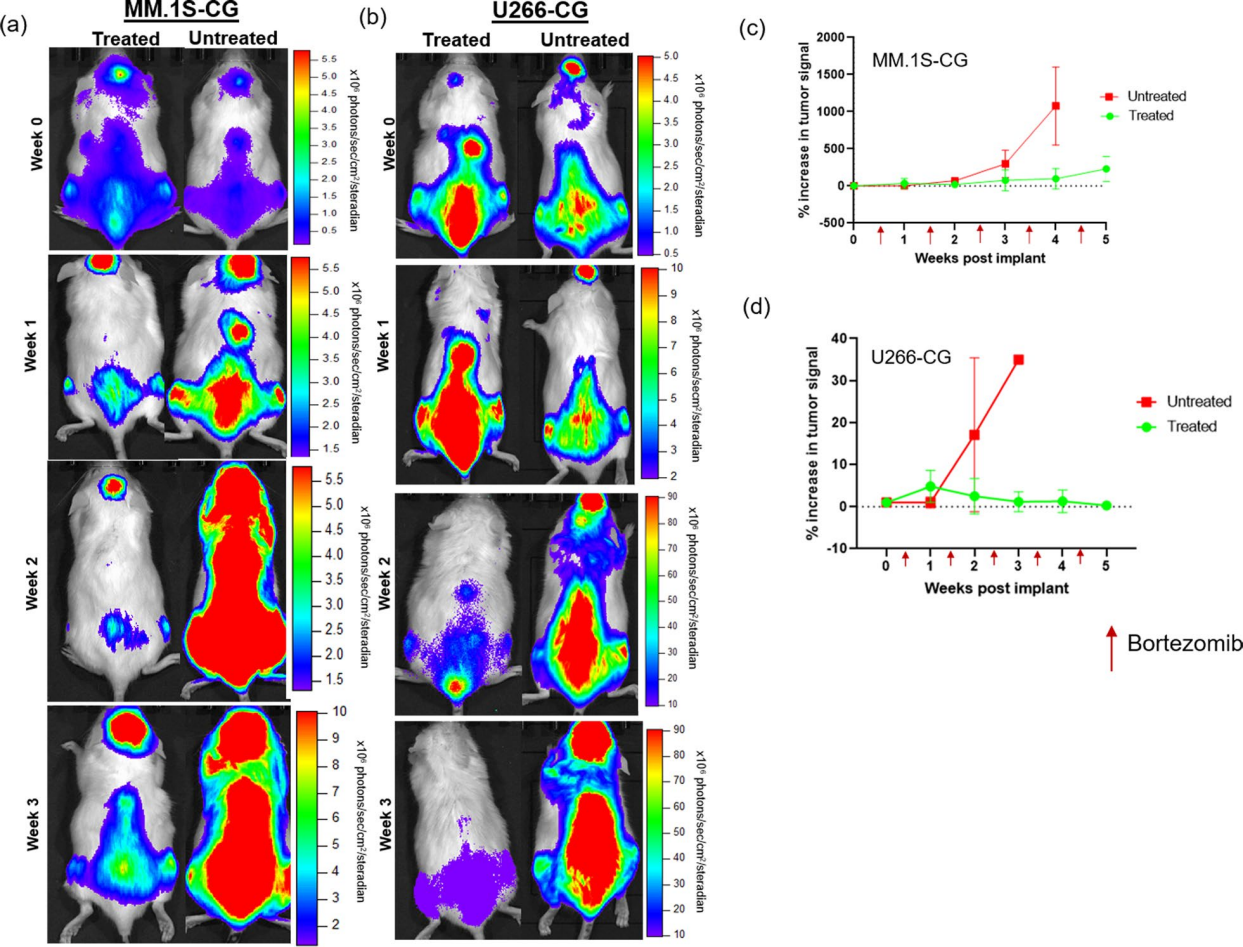
**a**, **b** Representative BLI images of mice injected with MM.1S-CG and U266-CG cells, respectively (treated and untreated cohorts), showing systemic tumor progression and response to bortezomib therapy over time. **c**, **d** Percent increase in bioluminescence signal intensity showing tumor progression in treated and untreated cohorts plotted as a function of time (weeks post-tumor implantation)

Small animal BLI was crucial for validating the establishment of the disseminated disease and for the longitudinal monitoring of the systemic disease progression in the MM.1S-CG and U266-CG disseminated (intravenous) tumor models. BLI images confirmed the presence of tumor in the bone marrow rich sites such as the spine, long bones (femur and tibia) and pelvis. Representative BLI images from the MM.1S-CG (Fig. 2a) and U266-CG (Fig. 2b) tumor bearing mice, showed increasing signal in no treatment cohort; especially in the spine and femurs as compared to the mice treated with bortezomib. In the MM.1S-CG myeloma mouse model (Fig. 2a), the BLI signal in both the treated and untreated mice showed similar tumor burden at the baseline imaging time point (week 0; defined as the week prior to the start of bortezomib therapy). Bortezomib treatment was started post-baseline imaging (i.e., after week 0). Quantitative and qualitative BLI signal showed significant percent increase (*p* < 0.05) in tumor burden in mice with no treatment as compared to the ones treated with bortezomib over the period of time (week 0 to week 4) (Fig. 2c). Post-initiation of bortezomib therapy at week 4, the percent increase in tumor signal increased more than tenfold in untreated mice as compared to the treated mice.

Similar to MM.1S-CG, the BLI data for U266-CG tumor bearing mice (Fig. 2b) demonstrated the expected pattern of tumor engraftment confirming comparable tumor burden in both treated and untreated mice at week 0 (i.e., prior to start of treatment). The BLI signal in bortezomib treated mice decreased with time; whereas in the no treatment mice cohort, the BLI activity increased significantly post-week 3 of tumor inoculation.

### Small animal PET imaging

**Fig. 3:**
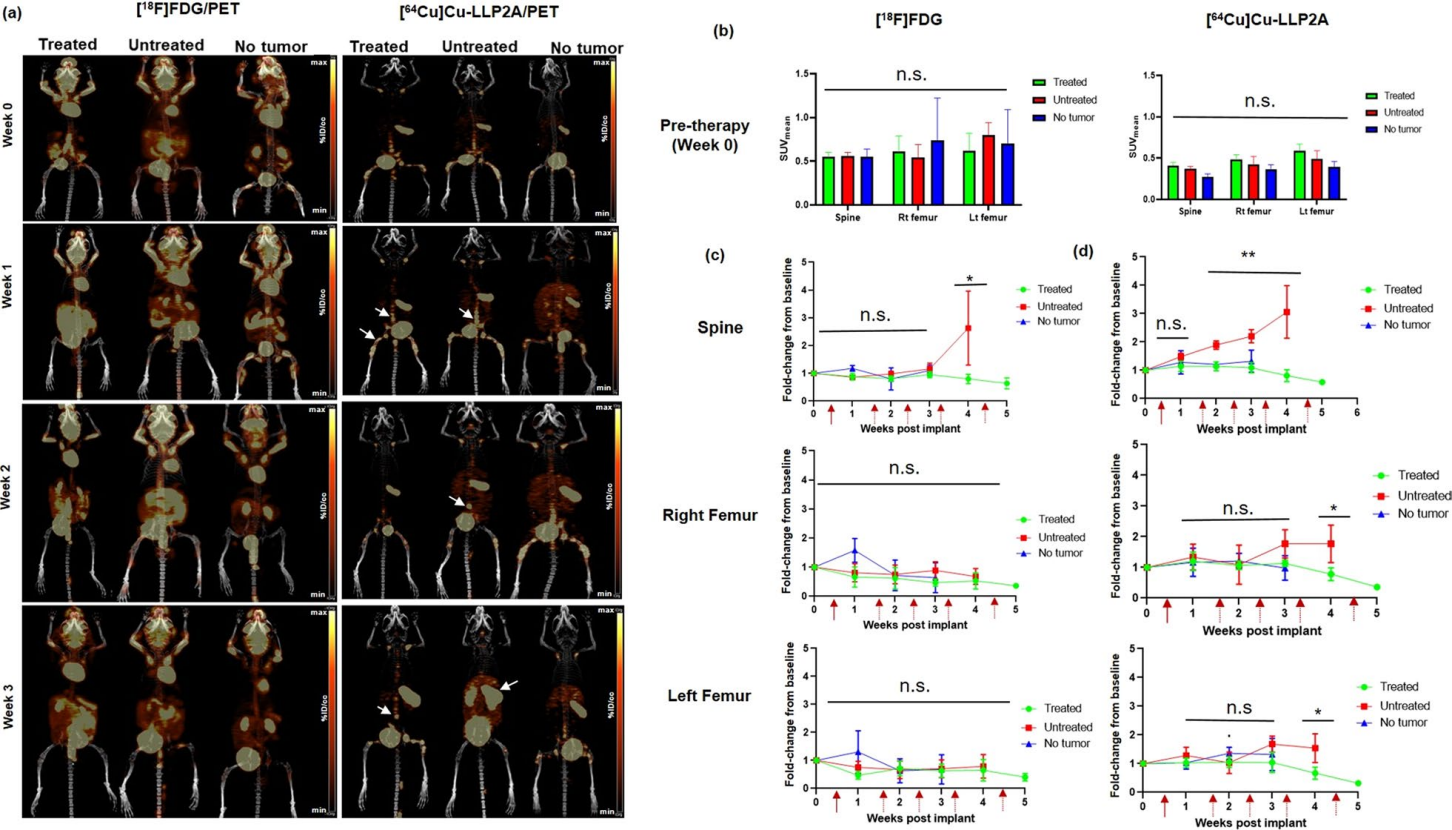
**a** Representative small animal maximum intensity projection (MIP) [^18^F]FDG and [^64^Cu]Cu-LLP2A PET images in disseminated MM.1S-CG tumor mice (treated and untreated) as well as in no tumor mouse cohorts, respectively. Bortezomib therapy was started in the treatment cohort between weeks 0–1. White arrows are pointing to the selected focal tumor lesions in the treated and untreated mice imaged with [^64^Cu]Cu-LLP2A/PET. Scale Bar ([^18^F]FDG): Min 4% ID/cc, Max 8% ID/cc; Scale Bar ([^64^Cu]Cu-LLP2A): Min 3% ID/cc, Max 6% ID/cc. **b** [^18^F]FDG and [^64^Cu]Cu-LLP2A PET quantification (SUV_mean_) of the bone marrow rich organs – spine, right (Rt) femur and left (Lt) femur, in MM.1S-CG disseminated tumor model and no tumor mice at week 0 (baseline, before therapy was started). **c** [^18^F]FDG SUV fold-change from baseline in the spine, right (Rt) femur and left (Lt) femur in MM.1S-CG tumor (treated and untreated) mice and no tumor mice, plotted as a function of time. **d** [^64^Cu]Cu-LLP2A SUV fold-change from baseline in the spine, right (Rt) femur and left (Lt) femur in MM.1S-CG tumor mice (treated and untreated) and non-tumor mice, plotted as a function of time. *p ≤ 0.05, **p ≤ 0.01

Longitudinal small animal PET imaging study was performed in both the MM.1S-CG and U266-CG mouse models (treated and untreated cohorts) as well as in the non-tumor healthy control cohort. The goal was to compare the performance of the clinical gold standard metabolic radiotracer, [^18^F]FDG, with the VLA4 targeted imaging agent, [^64^Cu]Cu-LLP2A, in evaluating bortezomib therapy response in mice with MM.1S-CG and U266-CG disseminated myeloma disease. In the MM.1S-CG tumor bearing, treated and healthy control mice, [^18^F]FDG PET showed normal physiological uptake in the metabolically active organs such as brain, gastrointestinal tract and heart. [^18^F]FDG PET also showed background uptake in the leg muscles and brown fat (Fig. 3a). The images of [^64^Cu]Cu-LLP2A PET showed robust uptake of this radiotracer in the tumor rich skeletal sites such as spine, pelvis and femurs (confirmed by BLI), whereas mice with no tumor burden did not show significant [^64^Cu]Cu-LLP2A uptake in these sites (Fig. 3a). The SUV_mean_ [^18^F]FDG PET and [^64^Cu]Cu-LLP2A PET data at the pre-therapy imaging time point showed no significant differences in spine, right, and left femur uptake among the tumor bearing (mice divided into two groups—treated (bortezomib therapy) and untreated) and no tumor mice (Fig. 3b). Figure 3c shows the comparison of percent change in SUV_mean_ (fold-change from baseline) among treated, untreated and no tumor cohorts of mice with both [^18^F]FDG PET and [^64^Cu]Cu-LLP2A PET over a period of time (week 1–6). Bortezomib therapy was initiated post-baseline imaging, and then longitudinal imaging was done with both the tracers up to 6 weeks for treated mice and up to week 3 and 4 for no tumor and untreated mice, respectively (Fig. 1). The SUV_mean_ data from [^18^F]FDG PET imaging showed threefold increase in the spine uptake of untreated mice as compared to treated mice at week 4 (Fig. 3c). The SUV_mean_ uptake of [^18^F]FDG in both the femurs was comparable in all three groups throughout the study. The data from [^64^Cu]Cu-LLP2A imaging showed significant increase in spine uptake for the untreated group of mice as compared to the treated cohort (0.58 ± 0.13 vs 0.31 ± 0.05) at week 4. No significant changes were observed in the spine uptake among treated and no tumor mice. The right and left femurs started showing changes in the SUV_mean_ by week 4 between the treated and untreated cohorts with twofold increase in [^64^Cu]Cu-LLP2A uptake, respectively (Fig. 3c).

**Fig. 4:**
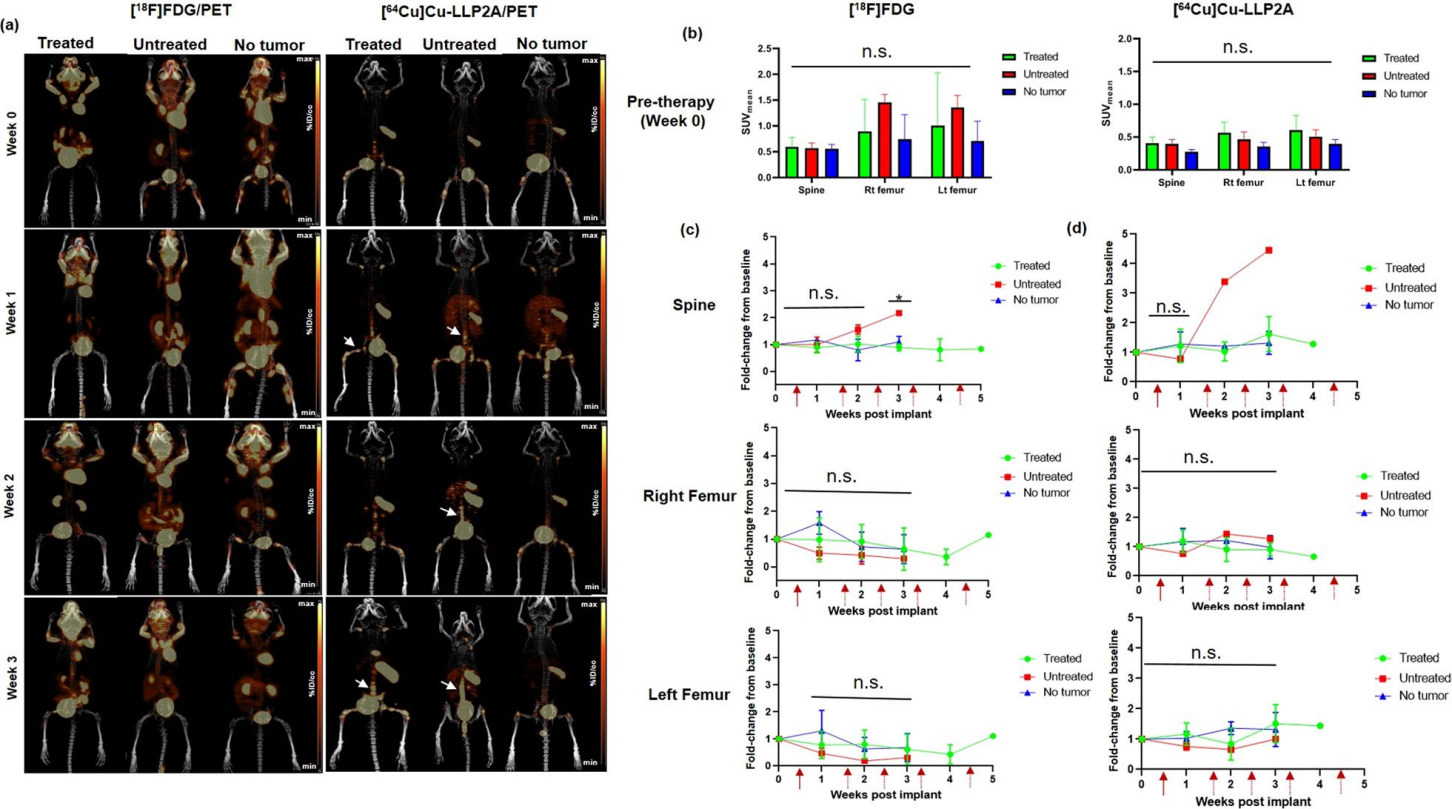
**a** Representative small animal maximum intensity projection (MIP) [^18^F]FDG and [^64^Cu]Cu-LLP2A PET images in disseminated U266-CG tumor mice (treated and untreated) as well as in no tumor mouse cohorts, respectively. Bortezomib therapy was started in the treatment cohort between weeks 0–1. White arrows are pointing to the selected focal tumor lesions in the treated and untreated mice imaged with [^64^Cu]Cu-LLP2A/PET. Scale Bar ([^18^F]FDG): Min 4% ID/cc, Max 8% ID/cc; Scale Bar ([^64^Cu]Cu-LLP2A): Min 3% ID/cc, Max 6% ID/cc. **b** [^18^F]FDG and [^64^Cu]Cu-LLP2A PET quantification (SUV_mean_) of the bone marrow rich organs—spine, right (Rt) femur and left (Lt) femur, in U266-CG disseminated tumor model and no tumor mice at week 0 (baseline, before therapy was started). **c** [^18^F]FDG SUV fold-change from baseline in the spine, right (Rt) femur and left (Lt) femur in U266-CG tumor (treated and untreated) mice and no tumor mice, plotted as a function of time. **d** [^64^Cu]Cu-LLP2A SUV fold-change from baseline in the spine, right (Rt) femur and left (Lt) femur in U266-CG tumor mice (treated and untreated) and no tumor mice, plotted as a function of time. *p ≤ 0.05, **p ≤ 0.01

Figure 4a shows representative maximum intensity projection (MIP) small animal PET images of [^18^F]FDG at 1 h and [^64^Cu]Cu-LLP2A at 4 h post-injection of the respective tracers in the U266-CG tumor bearing mice. [^64^Cu]Cu-LLP2A PET identified the tumor burden in spine and leg bones of tumor bearing mice while [^18^F]FDG showed background uptake in heart, brain, leg muscles and brown fat. [^18^F]FDG and [^64^Cu]Cu-LLP2A SUV data at week 0 confirmed comparable tumor burden in spine, right femur, and left femur of U266-CG tumor bearing mice (grouped as treated and untreated) and no tumor mice (Fig. 4b). The SUV_mean_ quantification and comparison data with [^18^F]FDG PET showed increased [^18^F]FDG uptake in spine of untreated mouse (SUV_mean_ of 0.93) which was almost twice as high as that of mice treated with bortezomib (SUV_mean_ 0.54 ± 0.16) at week 3 (Fig. 4c). Similarly, [^64^Cu]Cu-LLP2A PET data showed significantly higher SUV_mean_ uptake of 1.56 in spine of untreated mouse, as compared to 0.59 ± 0.27 in case of treated mice. However, both the femurs of untreated mice showed low [^18^F]FDG uptake (non-significant) as compared to the treated and no tumor mice. The comparison of SUVs from all the three groups (treated, untreated and no tumor age-matched) for right and left femurs using [^64^Cu]Cu-LLP2A showed no significant changes in the uptake of the radiotracers in these tissues.

### In vitro and ex vivo flow cytometry

**Fig. 5:**
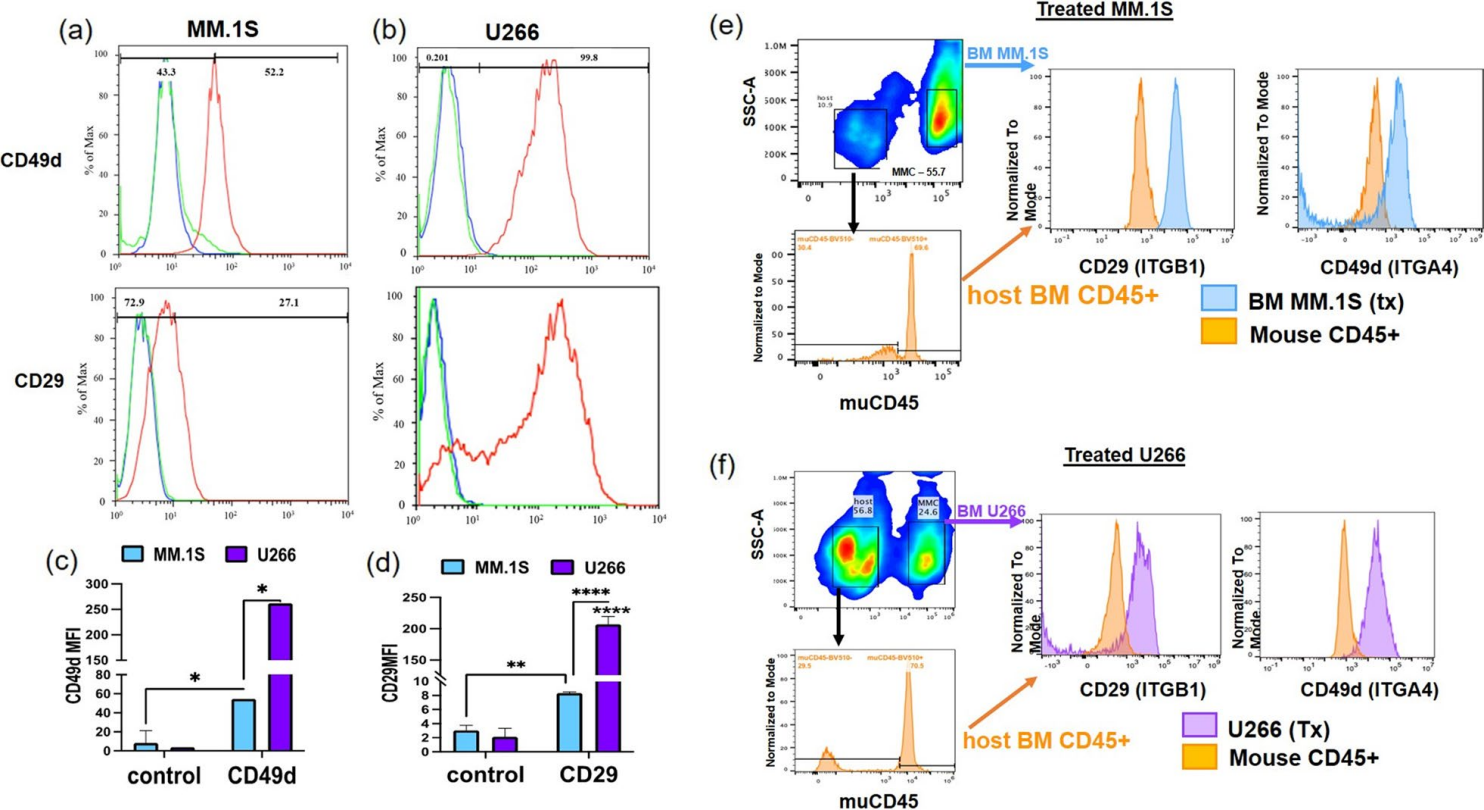
Representative CD49d/α_4_ (upper panel) and CD29/β_1_ (lower panel) surface expression levels in **a** MM.1S, **b** U266 cell lines analyzed by flow cytometry. **c** Comparison of CD49d mean fluorescence intensity (MFI) values among MM.1S and U266 cell lines. The non-specific surface binding was evaluated by isotype (IgG) controls. **d** Comparison of CD29 MFI values among MM.1S and U266 cell lines. Representative CD49d/α_4_ and CD29/β_1_ surface expression levels in BM cells of **e** MM.1S-CG cells injected mice, **f** U266-CG cells injected mice analyzed by flow cytometry

In vitro-cultured MM.1S and U266 cells were analyzed for surface expression of CD49d (α_4_ sub-unit of VLA4/α_4_β_1_) and CD29 (β_1_ sub-unit of VLA4/α_4_β_1_) using flow cytometry, which showed expression in both cell lines, albeit at different levels (Fig. 5a, b). The mean fluorescent intensity (MFI) of CD49d in U266 cells was significantly higher than the MM.1S cells. Similarly, the MFI values for CD29 were significantly higher in U266 cells as compared to MM.1S cells. Only 52% of MM.1S cells stained positive for CD49d and ~ 30% stained for CD29 (Fig. 5c, d). MFI values were normalized to the isotype control to account for the non-specific (IgG) surface binding.

Analysis of ex vivo bone marrow from the tibial, femoral, and pelvic bones showed the persistence of GFP/human CD-45 positive MMC in bortezomib treated mice. Gating of these tumor cells showed expression of human CD49d (α_4_) and CD29 (β_1_), while bone marrow of mouse CD45 + cells remain unstained (Fig. 5e, f). This indicates that, in vivo, U266-CG and MM.1S-CG cells surviving therapy with bortezomib may still be detected by VLA4 imaging.

## Discussion

Imaging plays an indispensable role in the drug development process as well as in translation of preclinical findings into patient care [53]. Hematological malignancies stand to greatly benefit from molecularly targeted whole-body advanced imaging methods. Recently, there have been exciting developments in the sphere of MM imaging. Non-invasive imaging modalities such as PET/CT or PET/MRI have been extensively used to investigate bone lesions and metastasis in MM [26, 54, 55]. Our group has previously showed that antibody-based PET nuclear agents, [^89^Zr]Zr-daratumumab and [^89^Zr]Zr-elotuzumab targeted to overexpressed proteins CD38 and CS1, respectively, on myeloma cells can be used as companion diagnostics for preclinical imaging of MM [26, 54]. Ulaner et al. demonstrated the first-in-human use of [^89^Zr]Zr-daratumumab in measuring MM burden, evaluating minimal residue disease (MRD), and assessing daratumumab therapy response [56]. PET imaging with CXCR4 targeted [^68^Ga]Ga-Pentixafor, another promising radiotracer, enabled detection of myeloma lesions that could not be detected with [^18^F]FDG and paved way for selecting patients for CXCR4 directed therapies [29, 55].

[^18^F]FDG is the gold standard MM imaging agent for MM patient management largely due to its ability to differentiate between metabolically active and inactive sites [57–59]. While used prolifically, [^18^F]FDG has limitations in therapy response monitoring in MM patients. PET imaging using [^18^F]FDG can lead to false positive signal due to increased uptake in a treatment setting due to therapy related flare[60], and false negatives due to decreased uptake in hypometabolic MM lesions [26, 61]. To overcome these limitations, we propose the use of alternative tracers to evaluate therapy response in MM.

The activated conformation of VLA4 (also known as the α_4_β_1_ integrin) is overexpressed in MM and plays an important role in myeloma cell homing, adhesion and survival within the bone marrow [62]. Previous studies have shown that ^64^Cu labeled high-affinity peptidomimetic ligand, LLP2A ([^64^Cu]Cu-LLP2A) can be used to target VLA4 in murine melanoma mouse model and can efficiently detect small metastatic lesions [36]. Another recently published study demonstrated the use of [^64^Cu]Cu-LLP2A as a PET imaging agent to detect VLA4-mediated hyper adhesion in transgenic sickle cell disease mice [63]. Our group has previously evaluated the potential of [^64^Cu]Cu-LLP2A to image VLA4 expression in MM mouse models [31, 35]. In this study, we sought out to evaluate the efficacy of the metabolic tracer [^18^F]FDG and VLA4 targeted [^64^Cu]Cu-LLP2A for monitoring bortezomib (proteasome inhibitor) therapy response using longitudinal small animal PET imaging.

Two human myeloma cell lines, MM.1S and U266, with variable VLA4 surface expression were chosen for the study to assess any identifiable imaging signatures likely due to VLA4 expression on the malignant plasma cells. To further emulate medullar myeloma disease, disseminated myeloma was induced in NOD-SCID Gamma (NSG) mice by injecting MM.1S-CG and U266-CG cells via tail vein, and the tumor progression was monitored weekly by BLI. Bioluminescence imaging (BLI) confirmed the presence of tumor in the bone marrow rich skeletal sites such as spine, pelvis and leg bones. Bioluminescence imaging (BLI) data also showed that the tumor distribution in vivo was not uniform among the various skeletal sites. We postulated that the observed differential tumor burden in vivo could be because of the heterogeneous nature of the disseminated myeloma disease. The FDA approved reversible proteasome inhibitor, bortezomib, which is widely used either alone or in combination with other agents for treating MM, was our therapy of choice. The comparison of BLI data between treated and untreated cohorts of mice with MM.1S-CG and U266-CG tumors showed decreasing light signal in mice treated with bortezomib as compared to tumor bearing mice with no treatment.

Longitudinal small animal PET imaging with [^18^F]FDG and [^64^Cu]Cu-LLP2A, respectively, was sequentially performed to evaluate bortezomib therapy response in these two human MM models. [^18^F]FDG small animal PET/CT imaging at 1 h post-injection of the radiotracer showed background physiological uptake in the brain, GI tract, brown fat and leg muscle [64]. [^18^F]FDG is a biomarker for glucose metabolism. It requires GLUT1 protein on the cell surface for the internalization and hexokinase-2 enzyme for retention in tumor cells; but the expression of GLUT 1 transporters and hexokinase 2 is inconsistent in MM cells [27, 65, 66]. In both the MM.1S and U266 tumor models, no significant changes in [^18^F]FDG uptake were observed in the right and left femurs among the bortezomib treated, untreated, and no tumor cohorts. However, [^18^F]FDG PET started to show significant difference in spine SUV among the treated and untreated cohorts at week 4 in MM.1S-CG and at week 3 in U266-CG myeloma mice.

The SUV_mean_ data from [^64^Cu]Cu-LLP2A PET imaging showed that VLA4 targeted [^64^Cu]Cu-LLP2A could detect changes in the tumor burden in spine as early as week 2 among the treated, untreated and no tumor cohorts. The femurs of MM.1S-CG tumor bearing mice, with no treatment, showed comparatively increased uptake at week 4 as compared to the MM.1S-CG tumor bearing mice treated with bortezomib. Previous studies have showed that bortezomib downregulates VLA4 expression in myeloma cells [43]. This is consistent with reduced translation of membrane proteins during proteotoxic stress. A later study, however, suggested that myeloma cells resistant to bortezomib may increase expression of VLA4 and its function in vivo [44]. In the femurs of U266-CG tumor bearing mice, however, there were no significant differences. This aligned with the BLI data that showed that majority of tumor burden was concentrated in the spine. The PET SUV data of spine for U266-CG mice (treated and untreated) demonstrated similar trend as that of BLI signal of spine in these mice. [^64^Cu]Cu-LLP2A uptake was comparable between the treated and no tumor cohorts in both the mouse models. Finally, [^64^Cu]Cu-LLP2A binds to the activated form of VLA4, and normal cells such as leukocytes [67]. In this study, we utilized immunosuppressed mouse models of human myeloma to focus primarily on the MM cells in the bone marrow. The contribution of the inflammatory cancer milieu in the bone marrow toward VLA4 uptake has been addressed previously in the immunocompetent model of mouse myeloma (5TGM1/KaLwRij)[35].

## Conclusion

This study demonstrated significant qualitative and quantitative differences in the uptake of standard-of-care [^18^F]FDG and VLA4 targeted [^64^Cu]Cu-LLP2A tracers in human models of disseminated MM. [^64^Cu]Cu-LLP2A PET detected changes before [^18^F]FDG PET. [^18^F]FDG signal was observed in the brain, gut and heart, whereas the primary MM disease in the spine and long bones was poorly visible. Based on the in vitro*, *ex vivo and in vivo data, we posit that [^64^Cu]Cu-LLP2A is a promising PET imaging agent for treatment monitoring in MM patients. The imaging approach can be further extended to stratify patients for specific therapies.

## Supplementary Information

**Additional file1. Supplementary Fig S1:** (a) Radio-HPLC chromatogram of [^64^Cu]Cu-LLP2A showing > 99% of radiolabeled product at the retention time of 5.6 min. (b) UV-visible spectrum showing the LLP2A peak at the same retention time as that of radiolabeled LLP2A. **Supplementary Fig S2:** Kaplan-Meier survival plots of MM.1S-CG and U266-CG disseminated tumor bearing NOD-SCID Gamma (NSG) mice (treated n=6/group and untreated n=6/group). **Supplemtary Fig S3: Representative Images.** (a) Region of Interest (ROI) drawn on a whole body of a mouse to determine the BLI signal. (b) ROI drawn on PET/CT slices on the sagittal sections for spine, right and left femur.

## Data Availability

All data generated and analyzed during the current study are included in this main article and its supplementary information files.

## Abbreviations

MM: Multiple myeloma
VLA4: Very Late Antigen-4
BM: Bone Marrow
[^18^F]FDG: Fluorine-18-fluorodeoxy glucose
[^64^Cu]Cu-LLP2A: Copper 64 labeled precursor LLP2A-CB-TE1A1P
NSG: NOD-SCID Gamma
PET: Positron Emission Tomography
IMWG: International Myeloma Working Group
MRD: Minimal Residual Disease
GLUT1: Glucose Transporter Protein 1
FDA: US Food and Drug Administration
CAM-DR: Cell Adhesion Mediated Drug Resistance
SUV_mean_: Mean Standard Uptake Value
BLI: Bioluminescent Imaging
ROI: Regions of Interest
MFI: Mean Fluorescent Intensity
