# Supplementary material for: In vivo quantitative assessment of therapeutic response to bortezomib therapy in disseminated animal models of multiple myeloma with [18F]FDG and [64Cu]Cu-LLP2A PET
Source: EJNMMI Res. 2021 Sep 29;11:97. doi: 10.1186/s13550-021-00840-4 (PMC8481408; doi:10.1186/s13550-021-00840-4)
Supplement: Supplementary file 1 — Additional file1. Supplementary Fig S1: (a) Radio-HPLC chromatogram of [64Cu]Cu-LLP2A showing > 99% of radiolabeled product at the retention time of 5.6 min. (b) UV-visible spectrum showing the LLP2A peak at the same retention time as that of radiolabeled LLP2A. Supplementary Fig S2: Kaplan-Meier survival plots of MM.1S-CG and U266-CG disseminated tumor bearing NOD-SCID Gamma (NSG) mice (treated n=6/group and untreated n=6/group). Supplemtary Fig S3: Representative Images. (a) Region of Interest (ROI) drawn on a whole body of a mouse to determine the BLI signal. (b) ROI drawn on PET/CT slices on the sagittal sections for spine, right and left femur. [file 13550_2021_840_MOESM1_ESM.pdf]

In vivo quantitative assessment of therapeutic response to bortezomib therapy in disseminated animal models of multiple myeloma with [^{18}F]FDG and [^{64}Cu]-CuLLP2A PET

Anchal Ghai[1], Nikki Fettig[1], Francesca Fontana[2], John DiPersio[3], Mike Rettig[3], Julie O Neal[3], Samuel Achilefu[1, 4, 5], Kooresh I. Shoghi[1], Monica Shokeen[1, 4*]

[1]Department of Radiology, Washington University School of Medicine, St. Louis, MO

[2]Department of Internal Medicine, Washington University School of Medicine, St. Louis, MO

[3]Department of Medicine, Washington University School of Medicine, St Louis, MO

[4]Department of Biomedical Engineering, Washington University in St. Louis, St. Louis, MO

[5]Department of Biochemistry and Molecular Biophysics, Washington University School of Medicine, St. Louis, MO

*Corresponding author: Monica Shokeen, Department of Radiology, Mallinckrodt Institute of Radiology, 4515 McKinley Avenue, 2nd floor, St Louis, MO 63110. Email: shokeenm@wustl.edu

Methods:

Quality control of $[^{64}Cu]$Cu-LLP2A

VLA4 targeted peptide, LLP2A-CB-TE1A1P (LLP2A), was radiolabeled with copper 64 (^{64}Cu). Ammonium acetate (0.1M; pH 5.5) was used as the radiolabeling buffer. LLP2A precursor (2.5 µg; 1.61 nmol) was incubated with **$[^{64}Cu]$Cu-Chloride** (74 MBq) for 30-40 min at 70°C with slight shaking. The radiolabeling efficiency was determined by high performance liquid chromatography (HPLC) using XB-C18 Kinetex column (Fig S1). 0.1%TFA in water and 0.1% TFA in acetonitrile were used as aqueous and organic mobile phase solvents.

Survival study

NOD SCID Gamma (NSG) mice were injected with MM.1S-CG and U266-CG human myeloma cell lines *via* tail vein. The tumor burden in these mice was evaluated weekly by BLI. The tumor bearing mice were divided into two cohorts where, one group was treated with bortezomib (1mg/kg; intraperitoneal injections) twice a week, and the mice from second group did not receive any treatment. MM.1S-CG and U266-CG systemic tumors bearing mice were imaged longitudinally with $[^{18}F]$FDG and $[^{64}Cu]$Cu-LLP2A. These mice were observed daily for any weight loss or paralysis and were evaluated for therapy response and survival. Mice were euthanized with the onset of morbidity/paralysis as per institutional guidelines. Survival was assessed until week 5 post inoculation of tumors (Fig S2).

Regions of interest for PET images

CT and PET images acquired with $[^{18}F]$FDG and $[^{64}Cu]$Cu-LLP2A were co-registered on Inveon Research Workplace (IRW) software (Siemens Medical Solutions, Knoxville, TN). The volumetric regions of interest (ROI) were manually drawn using a 2-dimensional (for femurs) and 3-dimensional (for spine) tools on the sagittal attenuation-corrected (using CT anatomical guidelines) PET slices as shown in Fig S3b.

Supplemental figures:

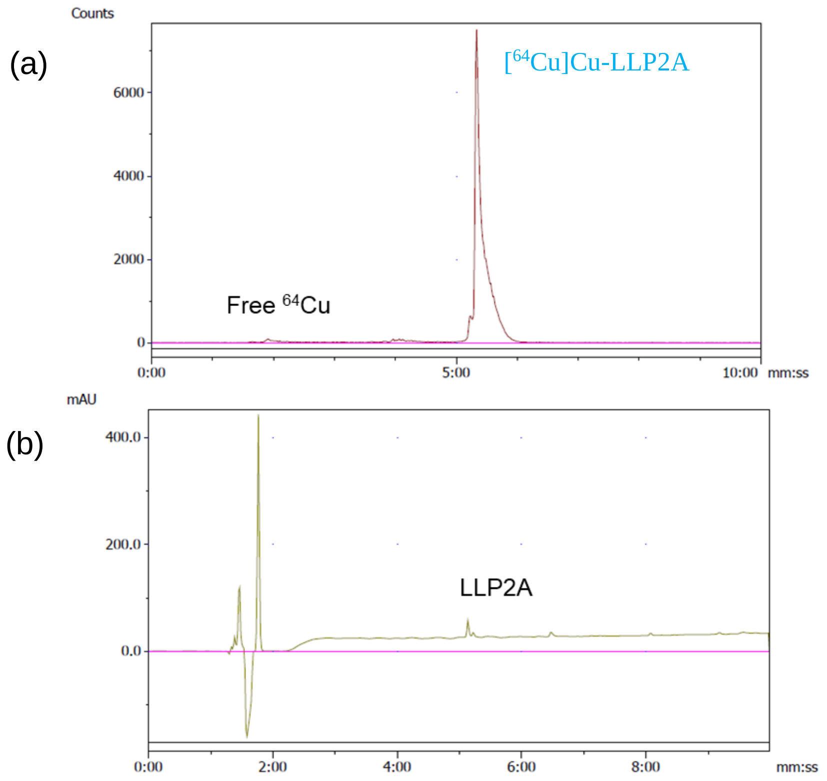

Fig S1 (a) Radio-HPLC chromatogram of $[^{64}Cu]$Cu-LLP2A showing > 99% of radiolabeled product at the retention time of 5.6 min. (b) UV-visible spectrum showing the LLP2A peak at the same retention time as that of radiolabeled LLP2A.

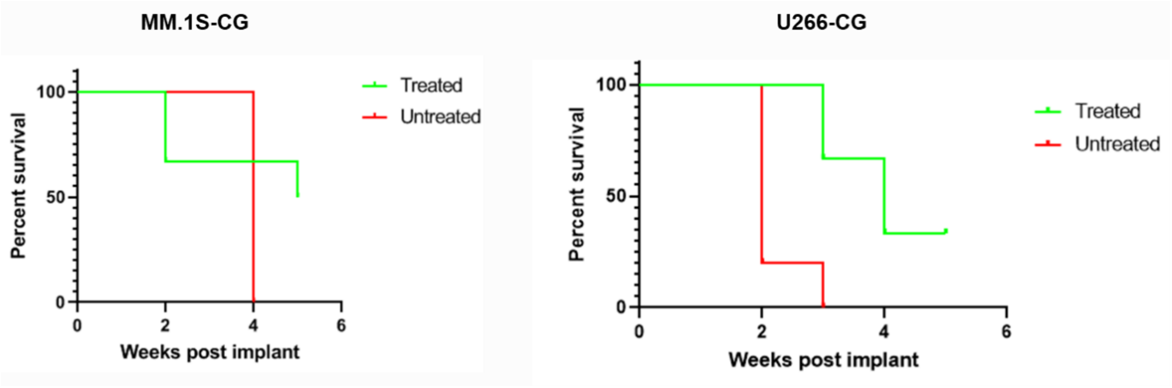

Fig S2 Kaplan-Meier survival plots of MM.1S-CG and U266-CG disseminated tumor bearing NOD SCID Gamma (NSG) mice (treated n=6/group and untreated n=6/group).

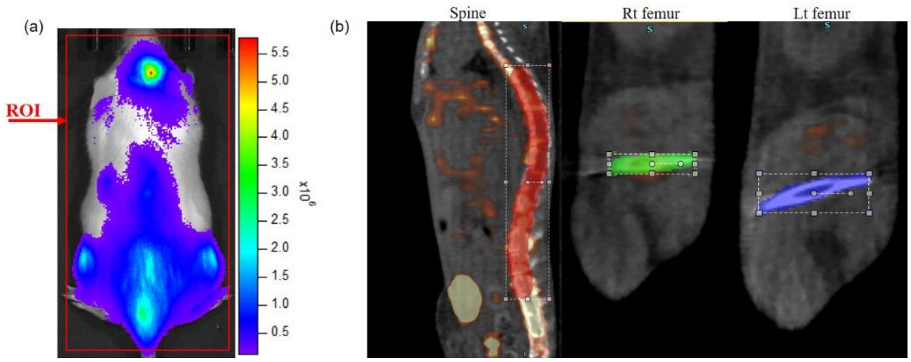

Fig S3 Representative Images. (a) ROI drawn on a whole body of a mouse to determine the BLI signal. (b) ROI drawn on PET/CT slices on the sagittal sections for spine, right and left femur.
